# Synthetic hydroxyapatite: a perfect substitute for dental enamel in biofilm formation studies

**DOI:** 10.1038/s41598-025-25634-9

**Published:** 2025-11-29

**Authors:** Johanna Dudek, Thomas Faidt, Claudia Fecher-Trost, Sudharshini Thangamurugan, Pardis Bayenat, Simone Trautmann, Jens Neurohr, Anne Holtsch, Frank Müller, Markus R. Meyer, Volkhard Helms, Karin Jacobs, Matthias Hannig

**Affiliations:** 1 Saarland University, Clinic of Operative Dentistry, Periodontology and Preventive Dentistry, Homburg/Saar, Germany; 2 Experimental Physics and Center for Biophysics, Saarland University, Saarbrücken, Germany; 3 Department of Experimental and Clinical Pharmacology and Toxicology, Center for Molecular Signaling (PZMS), Saarland University, Homburg, Saar Germany; 4 Center for Bioinformatics, Saarland University, Saarbrücken, Germany

**Keywords:** Biotechnology, Microbiology, Dentistry

## Abstract

In contact with saliva, tooth enamel is covered by biomolecules forming an initial biofilm. Microorganisms attach to the initial biofilm and form the bacterial biofilm, which can provoke diseases. Therefore, dental biofilms are the focus of preventive research. Enamel consists mainly of hydroxyapatite (HAP). Yet, the composition of dental apatite differs between individuals and influences enamel properties. Standardized surfaces might therefore be useful for biofilm research. Synthetic enamel-like HAP pellets perfectly meet the criteria for such well-defined samples. However, systematic investigations of synthetic HAP on oral biofilm formation have never been performed, especially not in comparison to enamel. Therefore, we systematically compared the in situ biofilm formation on synthetic HAP and enamel to investigate the suitability of HAP as a substitute for natural enamel in biofilm formation studies. We observed no differences in formation kinetics, microstructure and subject-specificity of the initial biofilm on both materials. Furthermore, at the proteome level the development of the biofilm on HAP follows the formation patterns observed for enamel. Formation kinetics and morphology of the bacterial biofilm were also subject-dependent and not distinguishable between the two materials. However, the bacterial viability on HAP was higher than on enamel. For bacterial biofilm viability studies, synthetic HAP may therefore even be the preferred substrate as it is more beneficial for identifying antimicrobial agents. In summary, the results prove synthetic HAP as perfect substrate for dental biofilm studies.

**Supplementary Information:**

The online version contains supplementary material available at 10.1038/s41598-025-25634-9.

## Introduction

Tooth enamel is the outer highly mineralized part of the dental crown. Humane mature enamel is composed of 95–97% hydroxyapatite (HAP), Ca_10_(PO_4_)_6_(OH)_2_, by weight, with less than 1% organic material and up to 2–4% water. Small, varying amounts of trace elements such as carbonate, magnesium (Mg) and fluorine (F) are found in the apatite fraction reflecting individual health history and environmental exposures^1–4^. These variations in the chemical composition influence the properties of dental enamel in different ways, for instance F has an anti-cariogenic effect, whereas Mg promotes caries formation^1,5,6^.

All surfaces in the oral cavity are covered by a biofilm. On intraorally exposed enamel surfaces, the biofilm formation starts within seconds after oral hygiene by adsorption of salivary biomolecules. The resulting initial acellular biofilm, called acquired pellicle, is composed of salivary macromolecules like carbohydrates and lipids, but predominantly of proteins^7,8^. Several hundred different proteins were identified within a pellicle layer formed during three minutes of oral exposure^9^. After rapid formation of the first protein layers, by direct interactions between the salivary proteins and the oral surfaces, protein–protein interactions and the adsorption of micelle-like protein agglomerates take place^8^. These agglomerates significantly increase the thickness of the initial biofilm. This second phase of initial biofilm formation is characterized by the continuous adsorption of further macromolecules and reaches a plateau at 30–120 min^10^. When imaged by transmission electron microscopy (TEM), the two phases of initial biofilm formation appear as a primary basal, electron-dense layer directly superimposed on the surface and, as a secondary, globular outer layer with lower electron density^8^. The pellicle modulates interfacial events occurring at the interface between the oral environment and the tooth such as lubrication*,* tooth protection against erosive and abrasive challenges as well as bacterial adhesion^11,12^. The detailed understanding of pellicle formation as well as its targeted manipulation with regard to the improvement of its protective properties is in the focus of dental research.

In the course of time, planktonic microorganisms attach directly to the pellicle leading to the formation of a dental bacterial biofilm. It starts with the specific attachment of mostly coccoid bacteria known as early colonizers^13^. These directed interactions serve as the basis for further bacterial adhesions resulting in a structured three-dimensional network of microorganisms. Mature bacterial biofilms are highly complex multispecies ecosystems comprising hundreds of different microbial species^14,15^. Under unfavorable conditions, bacterial biofilms can develop into pathogenic biofilms and thus lead to the development of numerous diseases^16^. For example, in the presence of excess carbohydrates, carbohydrate-fermenting bacteria may cause caries, which is characterized by local demineralization of dental hard tissues, initially the enamel^13,16,17^. In addition, dysbiosis of dental biofilms contributes to development and progression of systemic diseases^16^. For this reason, controlling the development of oral biofilms is of great importance for oral health. The regular use of fluoride-containing agents is an important anti-cariogenic strategy in this regard since many decades^17,18^. In recent years, however, the interest in novel bioinspired approaches for the management of dental biofilm formation has markedly increased and is the object of numerous scientific studies^19–22^.

Due to the severely limited availability of healthy human teeth for experimental purposes, dental studies are predominantly conducted with alternative materials. Because of its similarity to human enamel, bovine enamel has been used as such a substitute for many decades, despite its natural variations in chemical composition^23–26^. However, well-defined standardized tooth-like surfaces would be a better choice. HAP pellets prepared by pressure-less sintering of precompacted HAP powder meet the criteria for such well-defined enamel-like model samples with highly reproducible properties^27^. Using these samples in in vitro studies, the time dependence of fluoride uptake in HAP and effects of fluoride treatment on acid resistance of HAP surfaces were determined^28,29^. Also the adhesion of bacteria like *Streptococcus mutans*, one of the main oral microorganisms causing caries, was investigated^30^. A further in vitro study addressed the suitability as enamel-like model surface by comparing the adhesion of *Staphylococcus aureus* on tooth enamel and HAP pellets. To closely mimic oral physiological conditions, also the effects of saliva on *S. aureus* adhesion were examined^31^. This is of particular importance, since saliva-coated HAP behaves very differently compared to uncoated HAP. Previous work by Clark et al. showed that saliva coating decreased the numbers of certain species bound to HAP, whereas the numbers of other species were enlarged^32^. However, a systematic investigation of synthetic HAP surfaces with respect to all stages of oral biofilm formation has never been performed under physiological conditions, especially not in direct comparison to natural enamel.

In this study, we systematically compared the dental biofilm formation in the mouth cavity on natural tooth enamel and on synthetic HAP pellets to determine whether hydroxyapatite can be considered as a full substitute substrate for enamel in this regard. The biofilm formation was studied for both, the initial proteinaceous acellular biofilm and the mature bacterial biofilm. The initial biofilm was examined with respect to formation kinetics, microstructure, thickness and proteome composition. The bacterial biofilm was evaluated by means of biofilm formation kinetics, morphology, substrate surface coverage and bacterial viability.

## Materials and methods

### Human subjects

In situ formed biofilm was collected from five volunteers (female and male). The subjects neither exhibited active dental caries, periodontal disease, gingivitis, nor any other dental disease potentially affecting the oral fluid composition and gave their informed consent to participate in this study. The study was performed in accordance with the Declaration of Helsinki. Biofilm collection protocols were approved by the medical ethic committee of the Medical Association of Saarland, Germany (proposal # 54/21, 2021).

### Enamel samples preparation

**Fig. 1:**
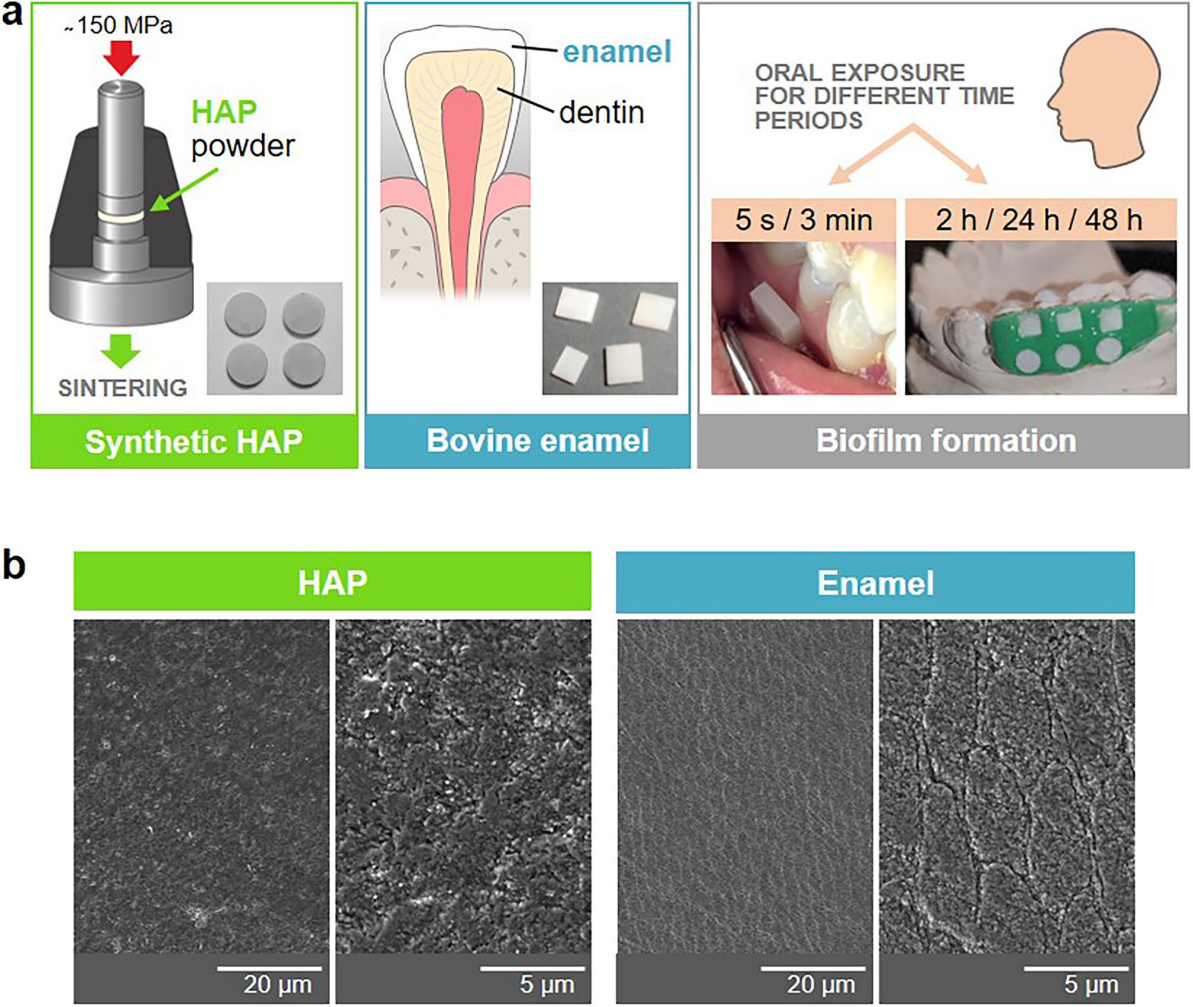
Synthetic HAP and natural enamel specimens used in this study. (**a**) Examples of ready-made HAP specimens sintered of HAP powder (left panel) and finally cut and polished enamel slabs from bovine incisor (middle panel). Examples of oral specimen exposure for biofilm formation (right panel). For 5 s and 3 min biofilm formation, HAP and enamel specimens were placed in the buccal vestibule of the lower jaw. For 2 h, 24 h and 48 h biofilm formation, the specimens were mounted on individual dental splints. Example of a subject-specific splint with mounted HAP and enamel specimens is shown. (**b**) The surface of ready for oral exposure HAP and enamel specimens (polished and purified) visualized by SEM microscopy.

Enamel slabs were prepared from the vestibular surfaces of bovine incisor teeth (Fig. 1a). The surfaces were progressively polished by wet grinding with up to 4000 grit (Buehler, Düsseldorf, Germany) and purified before oral exposure as described previously^10^.

### HAP samples preparation

The hydroxyapatite (HAP) pellets were made from HAP powder (Sigma-Aldrich, Steinheim, Germany) compressed in a stainless steel mold with a diameter of 5 mm (MsScientific, Berlin, Germany) and sintered according to the pressure-less sintering protocol described by Zeitz et al.^27^ (Fig. 1a). The final diameter of the specimens after sintering caused shrinking was about 4 mm. Before usage, the HAP samples were polished and purified in the same way as the enamel samples^10^. The HAP samples used had the same chemical composition, crystal structure, and surface roughness as specified by Zeitz et al.^27^.

### Biofilm formation

Thirty minutes before oral biofilm formation, tooth brushing without tooth paste and subsequent mouth rinsing with water was conducted by the volunteers. For 5 s and 3 min biofilm formation, HAP and enamel specimens were placed in the buccal vestibule of the lower jaw in the region of the premolar and molar teeth. For 2 h, 24 h and 48 h biofilm formation, the specimens were mounted on individual splints (Duran, SCHEU-DENTAL GmbH, Iserlohn, Germany) using silicone impression material (PRESIDENT light body, Coltène/Whaledent GmbH + Co. KG, Langenau, Germany) and exposed intraorally for the different time periods. During oral exposure, specimens were not subjected to any cleaning measures, furthermore volunteers generally refrained from using any tooth paste or any other agents for chemical cleaning procedure. During meals, splints were removed and stored in a wet chamber. After the intraoral exposure periods, specimens were prepared for analyses.

### Transmission electron microscopy (TEM)

The samples were washed with sterile water, fixed for 1 h at 4 °C with fixing solution (1% glutaraldehyde; 1% paraformaldehyde; cacodylate buffer: 0.1 M cacodylate/HCl pH 7.5), washed five times with cacodylate buffer for 10 min and contrasted with 2% osmium tetroxide in cacodylate buffer for 2 h. After washing five times with distilled water and dehydration in ethanol solutions with increasing concentrations (50%, 70%, 90%, 100%), the samples were incubated in acetone two times for 20 min, then in a mixture of Araldite CY212 (Agarscientific, Stansted, United Kingdom) and acetone (1:1) overnight, and finally embedded in Araldite at 65 °C overnight. After removing the enamel/HAP-part by decalcification in 1 M HCl for at least 3 h, the specimens were re-embedded in Araldite at 65 °C overnight. Then ultrathin-sections were cut in series with an ultramicrotome (Ultracut E, Reichert, Bensheim, Germany) using a diamond knife (Microstar 45°, Plano GmbH, Wetzlar, Germany), contrasted with 2% uranyl acetate for 10 min and lead citrate (pH 11.95) for 6 min, and analyzed with a TECNAI 12 BioTwin (FEI, Eindhoven, Netherlands).

### Scanning electron microscopy (SEM)

The samples were washed with sterile water, fixed with 2% Glutaraldehyde in 0.1 M cacodylate buffer/HCl pH 7.5 for 1 h at 4 °C, washed with cacodylate buffer and sequentially dehydrated in ethanol solutions with ascending concentrations (50–100%). After air-drying the specimens overnight, they were sputtered with carbon (Bal-tec *SCD* 030 *sputter* coater, Leica Microsystems, Vienna, Austria) and imaged at magnifications up to 20,000-fold using a Philips/FEI XL30 ESEM FEG microscope (FEI, Eindhoven, Netherlands).

### Atomic force microscopy (AFM) and surface properties analyses

For atomic force microscopy an Icon FastscanBio (Bruker-Nano, Santa Barbara, USA) was operated in peak force mapping mode in air using a Scanasyst-Air tip (Bruker-Nano) with a nominal spring constant of 0.4 N/m. Five random spots were scanned for each of three HAP and three enamel specimens with a scan area of 2 µm × 2 µm and a resolution of 1024 × 1024 pixels. All scans of the respective materials were used to analyze the surface properties root mean square (RMS) roughness and topography. Before calculating the average RMS roughness, a potential tilt of the surface was corrected by plain fitting. Since the RMS is not sufficient to have a proper topographical description of the surface the (averaged) Minkowski functional ${\widetilde{W}}_{1}$ (total three dimensional surface area, Table S1) was determined^33,34^. The functional was normalized by the size of the scan window (2 µm × 2 µm).

### Protein mass spectrometry (nanoLC-ESI-MS^2^) and raw data analysis

The preparation of the mass spectrometry (MS) samples was carried out as previously described^9^. Briefly, biofilms were collected at the same time for 3 min on HAP specimens or specimens consisting of enamel only, with a total surface area of 8 cm^2^. After washing with sterile water, the adsorbed material was chemically eluted from the specimens, precipitated and run on a NuPAGE Bis–Tris gel (Invitrogen, ThermoFisher Scientific). After subsequent trypsinization the resultant peptides were extracted and analyzed by nano liquid chromatography - electrospray ionisation - tandem MS (nanoLC-ESI-MS^2^). The mass spectrometric analysis was performed as previously described, with the exception of minor modifications^9^. Tryptic peptides were chromatographically separated for 160 min using a nano-UHPLC system (Ultimate 3000 RSLC) and analyzed with an LTQ Orbitrap Velos Pro mass spectrometer (ThermoFisher Scientific). Peptide and fragment masses were initially analyzed using Proteome Discoverer 1.4 (Thermo Fisher Scientific) and Mascot (Matrix Science, London, UK; version 2.7.0). Mascot was set up to search the SwissProtdatabase (release 2021_3, Homo sapiens, 20,396 entries) assuming the digestion enzyme trypsin, and searched with a fragment ion mass tolerance of 0.50 Da and a parent ion tolerance of 7.0 PPM. Carbamidomethylation of cysteine was specified as a fixed modification. Deamidation of asparagine and glutamine, oxidation of methionine and acetylation of lysine were specified as variable modifications. Dat-files were imported in the program Scaffold (Version 5.0, Proteome Software). Protein identifications were accepted if they had a probability greater than 95.0% (protein FDR ≤ 1.6% decoy) and contained at least 2 unique peptides/protein. Peptide identifications were accepted if they had a probability greater than 90.0% (peptide FDR ≤ 0.1% decoy) by the Scaffold Local FDR algorithm.

### Bioinformatics analysis of mass spectrometry data

The Pandas and NumPy Python libraries were employed to classify and sort the nano-mass spectrometry-generated proteomics data based on their adsorption to corresponding materials (HAP or enamel) and volunteers^35,36^. The Venn Python library was then used to visualize the number of proteins commonly adsorbed in all volunteers and the number of proteins uniquely adsorbed in each volunteer on each material as a Venn diagram^37^. The library was also used to visualize the number of proteins adsorbed on HAP and enamel from all volunteers (overlap) and from at least one volunteer (diversity). The Wilcoxon signed rank test was used to characterize the level of similarity of proteins adsorbed on HAP and enamel (supplementary information, Figure S2). We applied the null hypothesis, the number of shared proteins between the two HAP probes (or between two enamel probes) is equal to the number of shared proteins between one HAP and one enamel probe. Then, the number of proteins shared between HAP1 and HAP2 (or enamel 1 and enamel 2) shown in the Venn diagram was compared to the number of proteins shared between HAP and enamel. The *P*-value based on a threshold of 0.05.

### LIVE/DEAD® BacLight™ staining and fluorescence microscopy and statistics

The preparation of specimens for viability and coverage assessment was performed as previously described^38^. Briefly, samples were stained with SYTO 9 and propidium iodide containing solution (LIVE/DEAD® BacLight™ Bacterial Viability kit L7012, Invitrogen, Thermo Fisher Scientific, Carlsbad, USA) which stains living bacteria green and dead bacteria red, and examined using a fluorescence microscope (Axio Imager 2 Microscope, Zeiss MicroImaging, Göttingen, Germany). At least six representative randomized micrographs per specimen were taken at a magnification of 1000-fold using the image processing software AxioVision 4.8 (Carl Zeiss Microimaging, Göttingen, Germany). The biofilm colonization (coverage) and biofilm viability were evaluated by the image processing program ImageJ (ImageJ2, National Institutes of Health, LOCI, University of Wisconsin, USA). For the calculation of the coverage, all bacteria were considered, i.e. the sum of living and dead bacteria. The viability was calculated by subtracting the living bacteria from the total bacteria number. For statistical analyses of viability and coverage the mean values of all subjects (n = 5) were analyzed with the GraphPad Prism 10.3.1 software package (GraphPad Software). Normal distribution was verified using the Shapiro–Wilk test. The analysis was conducted by a two-tailed paired t-test. Statistical significance was set at *P* < 0.05.

### X-ray photoelectron spectroscopy (XPS)

For X-ray photoelectron spectroscopy, an ESCA Mk-II spectrometer with non-monochromatized Al-Kα radiation (photon energy 1486.6 eV) by Vacuum Generators was used. The tooth samples were probed in normal emission mode at pass energies of 50 eV (survey spectra) and of 20 eV (detail spectra) with step widths of 1.0 eV and 0.2 eV, respectively. For quantitative analysis of the elemental composition of the enamel samples, the detail spectra of O1s, Ca2p, Ca2s, P2p, P2s and F1s were corrected with a Shirley background and the peak intensities were scaled with the photoemission cross sections by Yeh and Lindau^39,40^. The base pressure during the XPS experiments (usually in the range of 10^−10^ mbar) increased to about 10^−6^ mbar due to a strong degassing of the samples.

## Results and discussion

For studying the formation of individual oral biofilms we used an in situ model, in which the test specimens from materials to be examined are placed in the oral cavity of the respective subject. For biofilm formation in a period of up to a few minutes, the specimens were placed directly in the buccal vestibule of the lower jaw. For longer biofilm formation periods of up to days, the specimens were mounted on subject-specific dental splints, which were then worn by the subjects to expose the specimens intraorally (Fig. 1a). Figure 1b illustrates SEM examinations of the specimens’ surfaces, HAP and enamel, after preparing them suitably (polished and purified) for oral exposure. The micrographs show a characteristic enamel surface with enamel rods consisting of tightly packed and regularly patterned apatite crystals. The surface of the HAP specimens is also of high density but shows different arrangement of the apatite crystals, which confirms our previous observations^27^. The surface properties of both materials were analyzed further by AFM (Fig. S1 and Table S1). The average RMS surface roughness was amounted to 15.6 ± 6.2 nm for HAP and 24.3 ± 5.3 nm for enamel (Fig. S1). The normalized and averaged surface area (in relation to 2 µm × 2 µm scan window) was calculated to (108 ± 6) % for HAP and (145 ± 16) % for enamel (Table S1), i.e. the relative accessible surface area for enamel is larger than the relative accessible surface area for HAP. However, the nano-scaled topography of both surfaces points to a similar adhesion behaviour of macromolecules forming the initial biofilm for both substrates.

### Initial biofilm: formation kinetics, microstructure and thickness

**Fig. 2:**
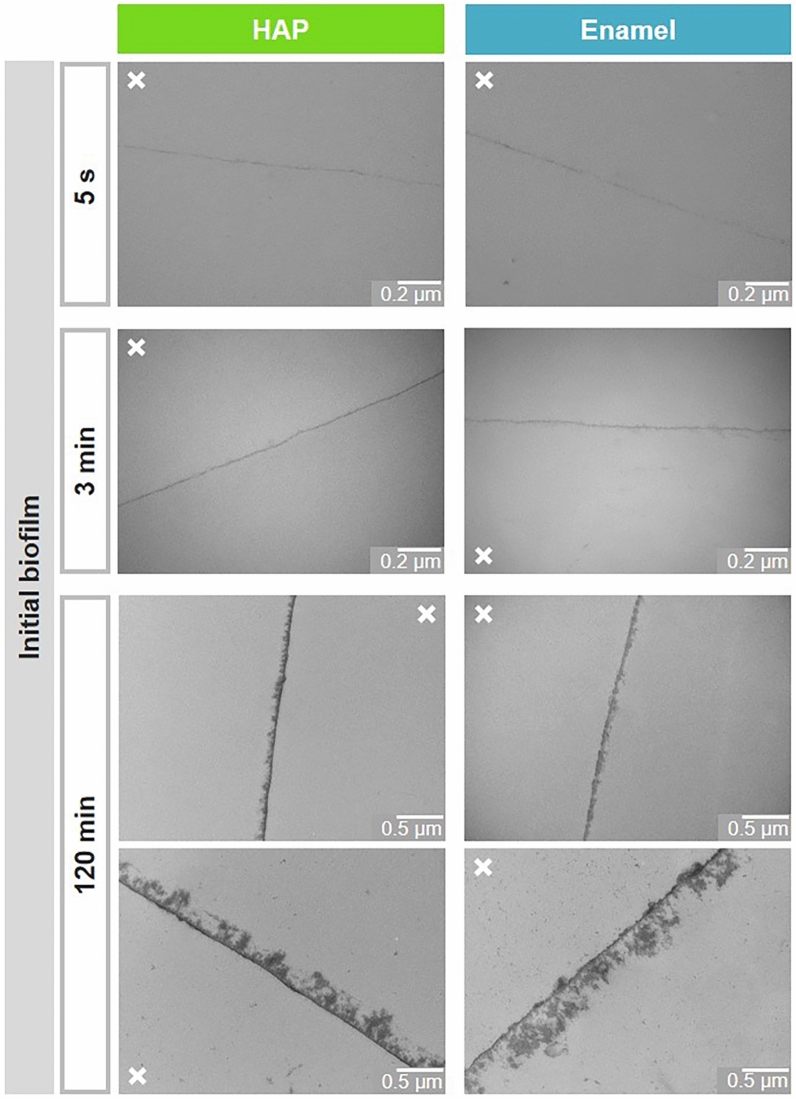
Representative TEM micrographs of the physiological initial 5 s, 3 min and 120 min biofilm formed on HAP or enamel. Note that HAP and enamel were removed during the preparation of the samples for TEM. The former HAP or enamel sites are marked with an x.

The development of the physiological initial biofilm formed in situ was visualized at 5 s, 3 min and 120 min after simultaneous intraoral exposure of HAP and enamel specimens by TEM (Fig. 2), respectively. As control, unexposed specimens were evaluated by TEM and did not show any deposits on both surface materials (data not shown). In contrast, an electron-dense almost continuous basal layer was observed already after 5 s of intraoral exposure on both substrates. Similar, slightly thicker homogenous pellicles in a low nanometer range were visible at the time point of 3 min on enamel as well HAP. Representative micrographs are shown in Fig. 2. After 120 min of biofilm formation in the oral cavity, loosely arranged globular conglomerates with lower electron density covered the electron-dense basal layer and the biofilm thickness increased, varying from a few 10 nm to some 100 nm. These observed differences in the biofilm thickness were independent of the substrate material but subject-specific. Examples of such 120 min biofilms are shown in Fig. 2 for two individual subjects (120 min; lower versus upper panels). Both the observed acquired pellicle thickness and formation kinetics as well as the inter-individual differences are in agreement with previous observations, demonstrating that the subjects have a decisive influence on these parameters^10^. Taken together, the thickness and microstructure of the initial biofilm on HAP follow the same formation patterns as on the enamel, with an electron-dense basal layer underneath an outer layer with lower electron density^8,41,42^.

### Initial biofilm: proteome

**Fig. 3:**
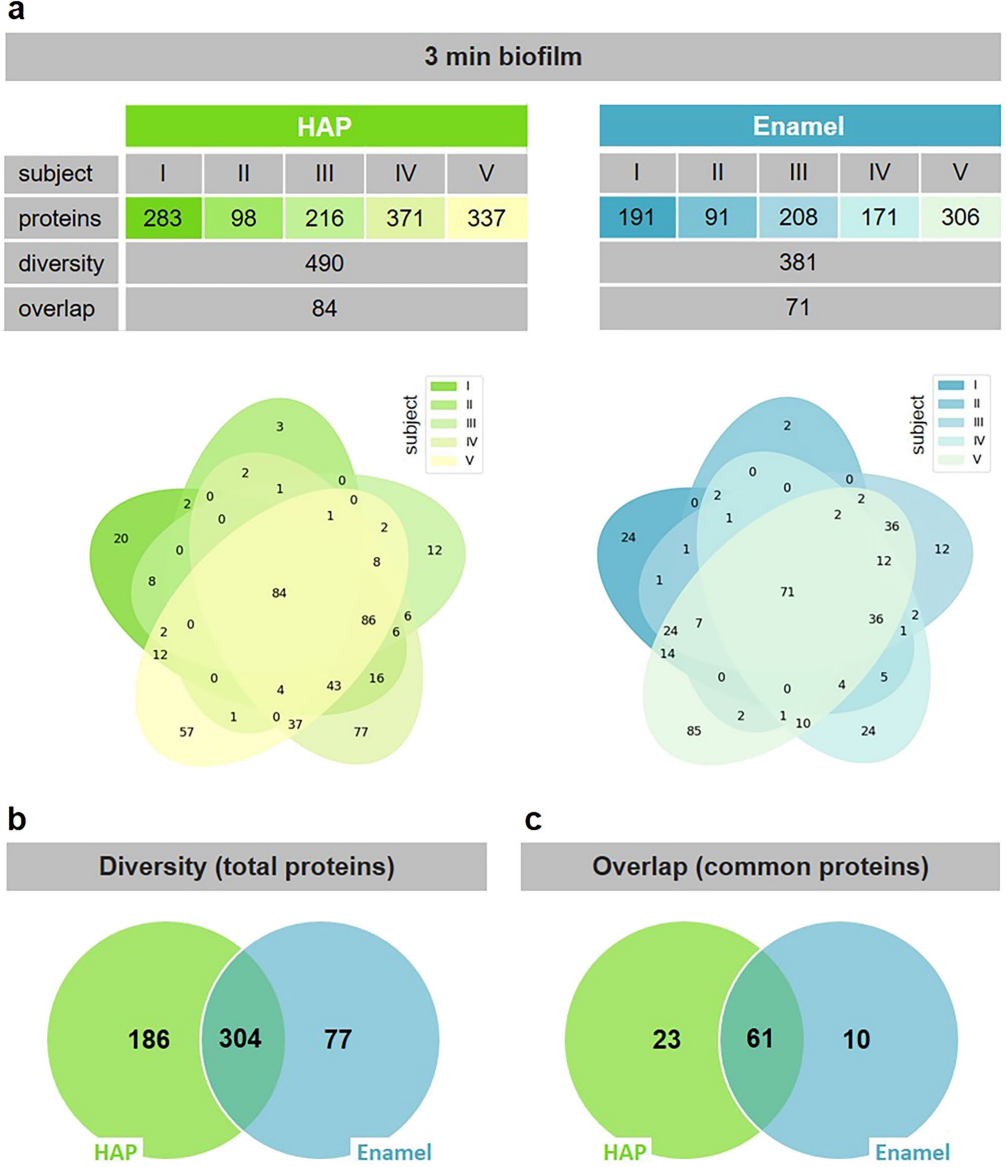
Proteome analysis of the 3 min initial biofilm formed on HAP or enamel as indicated. Pooled results of two independent oral exposure rounds (biological replicates) are shown. (**a**) Number of proteins in the biofilms of five subjects identified by protein mass spectrometry (nanoLC-ESI-MS^2^). Numbers inside the ellipses representing single subjects (I-V) indicate proteins identified within one or commonly within two to five subjects. (**b**) Total amount of identified individual proteins (diversity) in the biofilms of all subjects formed on HAP, enamel and both materials. (**c**) Number of identified common proteins (overlap) in the biofilms of all subjects formed on HAP, enamel and both materials.

The proteome of the initial biofilm was examined by protein mass spectrometry after chemical elution of a 3 min biofilm layer formed at the same time on both surface materials. The efficiency of the chemical elution was verified by TEM, which demonstrated an almost complete removal of the initial biofilm from both the HAP and the enamel surfaces (data not shown). Pooled results of two independent oral exposure rounds (biological replicates) are shown in Fig. 3a. Overall 567 different proteins were identified on both materials for all five subjects, 490 on HAP and 381 on enamel. Protein identifications in the same range were described before for enamel, but also for other dental materials such as ceramics, composite, gold or titanium^43,44^. On the individual subject level, the number of identified different proteins varied from 98 up to 371 on HAP. The overlap between individuals consisted of 84 common proteins. On enamel, the protein numbers identified in the initial biofilm of the five subjects differed from 91 up to 306. The overlap contained 71 common proteins (see also the Venn diagrams). Both the varying number of identified proteins on the individual subject level and the relatively small number of common proteins present on all subjects is in agreement with previously published studies, describing the pellicle proteome being an individual fingerprint^9,43,44^. In addition to the wide protein diversity, Trautmann et al. reported similar protein distribution patterns in the 3 min initial biofilm of individual subjects formed on enamel, with common 68 base proteins^45^. Here, a diversity of 304 proteins was found in the initial biofilm of the five subjects on both substrates (Fig. 3b). Furthermore, an overlap of 61 common proteins present on both materials was found in the initial biofilm of all subjects (Fig. 3c). Comparing the common 61 proteins to the base proteins of the 3 min initial biofilm described before, 84% identical or related proteins representing family members with similar properties and functions were found^45^. Considering the common proteins of the individual subjects, no significant differences between the materials were detected (Fig. S2). These results show a comparable protein composition of the initial biofilm on HAP and enamel. In summary, the development of the initial biofilm on HAP at the proteome level follows the formation patterns previously observed on dental enamel^45^.

### Mature biofilm: formation kinetics and morphology

**Fig. 4:**
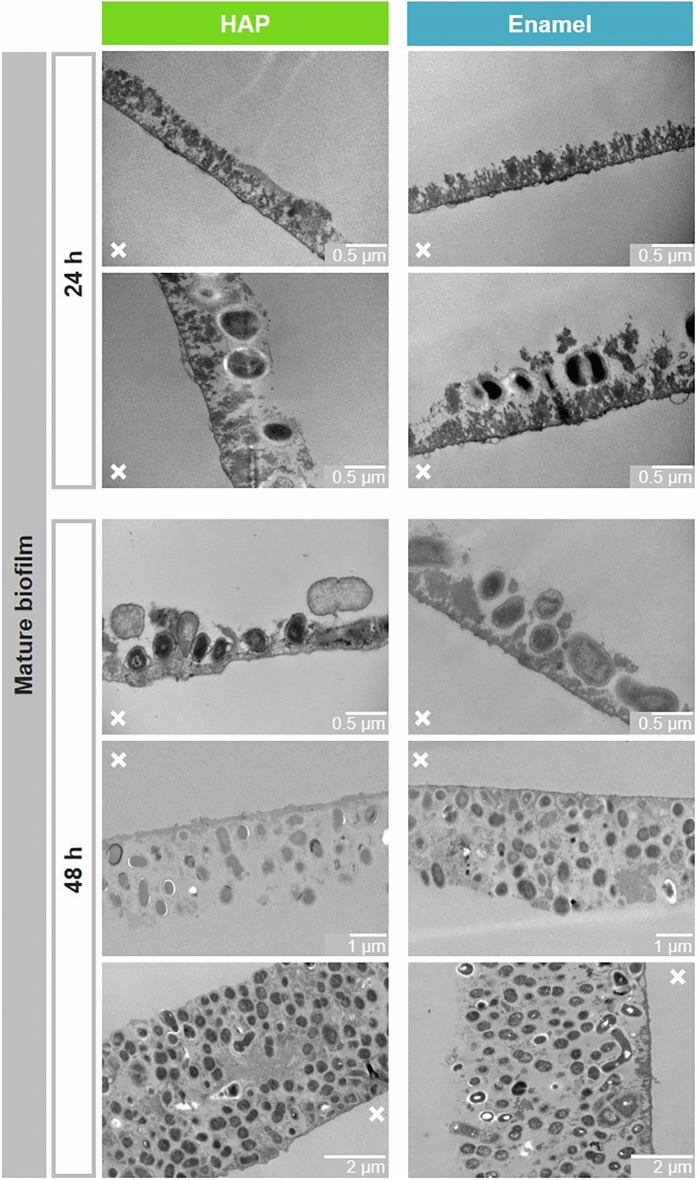
Representative TEM micrographs of the physiological mature 24 and 48 h biofilm formed on HAP or enamel. Note that HAP and enamel were removed during the preparation of the samples for TEM. The former HAP or enamel sites are marked with an x.

**Fig. 5:**
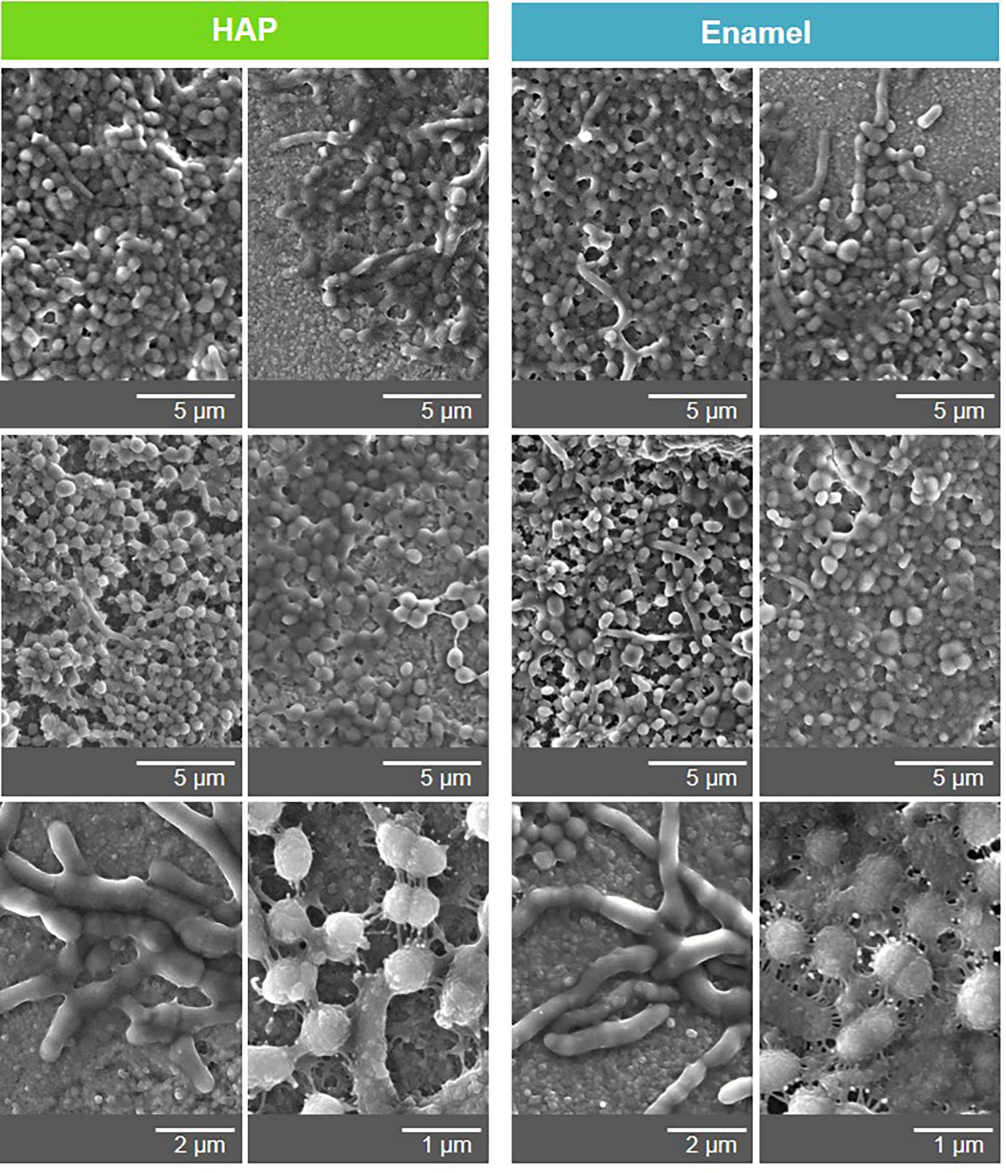
Representative SEM micrographs of the physiological mature 48 h biofilm formed on HAP or enamel.

During physiological biofilm maturation, microorganisms adhere to intraorally exposed surfaces. The mature bacterial biofilm was analyzed after 24 h and 48 h of oral exposure by TEM (Fig. 4). 24 h biofilms formed both on HAP and on enamel showed a further increase in deposits and the adhesion of first bacteria. Again subject-specific differences were detected (Fig. 4; 24 h: lower versus upper panels). The bacterial colonization continued during ongoing incubation in the oral cavity. The mature 48 h biofilms on both materials ranged subject-dependently from almost closed bacterial monolayers to complex multilayer bacterial consortia (Fig. 4). Additional SEM analysis of the 48 h biofilm formed on both materials confirmed these observations and showed surfaces coated with globular deposits covered by microorganisms ranging from monolayers to complex multilayer and multispecies biofilms (Fig. 5). Close contacts between the microorganisms and the deposits as well as between the individual microorganisms were visible within the complex mature biofilm. Furthermore, the SEM micrographs revealed in more detail the diversity of different types of bacterial species in the biofilms, e.g. cocci or rods. The bacterial diversity was subject specific, but independent of the substrate. Figure 5 depicts representative micrographs using examples of three subjects. These observations are consistent with bacterial composition studies by Tomás et al. on enamel, glass and HAP showing a distinct subject specificity in 48 h dental biofilms and significant differential abundance only in very few taxa of low abundance^46^. In summary, both analyses illustrate a characteristic development kinetics and morphology of the dental bacterial biofilm on both surfaces, HAP and enamel, with successional microbial colonization resulting in a complex multispecies microbial ecosystem^8,47^.

### Mature biofilm: bacterial coverage and viability

**Fig. 6:**
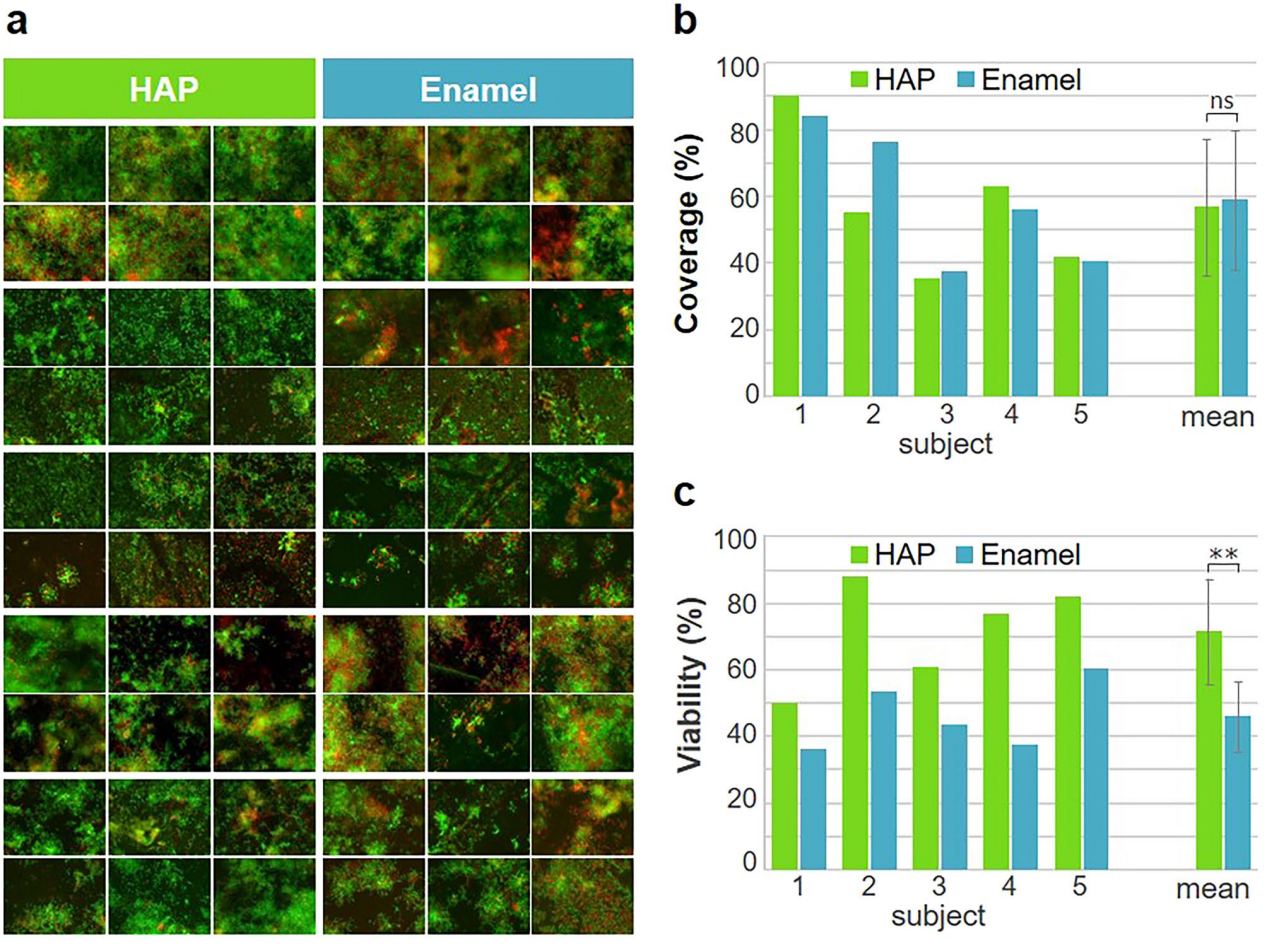
Bacterial coverage and viability: fluorescence micrographs of physiological mature 48 h biofilm formed on HAP or enamel. Each set of six shows representative micrographs from one subject for the respective materials. The micrograph sets of the respective subjects are arranged one below the other. The biofilm was stained with LIVE/DEAD® BacLight™ staining solution. (**a**) Living bacteria fluoresce green and dead bacteria red. (**b**) Quantification of biofilm coverage of each subject and the resulting mean values of all subjects. (**c**) Quantification of biofilm viability of each subject and the resulting mean values of all subjects. Error bars represent the standard deviation. ns: not statistically significant (*P* = 0.59), **: *P* = 0.0069.

The bacterial coverage and viability of a 48 h oral biofilm was analyzed by fluorescence microscopy after live/dead staining. Figure 6a shows the results for all subjects. The cellular coverage calculated from the total cells detected (living and dead) varied between the subjects and ranged from 36 to 90% on HAP and 37% to 84% on enamel (Fig. 6b). However, there were generally only minor differences in bacterial colonization between the two substrates. On average, the coverage with bacteria was 59% for HAP and 57% for enamel. In contrast to the coverage, the viability differed not only between the subjects but also between the substrates (Fig. 6c). The viability on HAP varied between the subjects from 36 to 60% and on enamel between 50 and 88%. The mean viability was 72% on HAP and 46% on enamel.

Of the trace elements found in tooth enamel, F has a negative effect on the bacterial metabolism^48^. Therefore, the F content in bovine enamel used in this study was determined by XPS. Fig. S3a depicts a typical XPS survey spectrum of enamel. Fig. S3b shows the relative amount of F in terms of atomic percent. When averaged over twelve teeth (taken from three cows), the mean value of the F amount is about 0.25 at-% (2500 ppm). The relative error of the F amount is about 25% of the mean value, which very well demonstrates the known variability in the chemical composition of natural enamel samples. In comparison there was no F detectable in the HAP samples. We can only speculate whether the amount of F detected in the enamel samples is indeed responsible for the increased mortality of bacteria on this material in the current study. However, previous studies detected an average amount of F on the enamel surface of 373 ± 235 ppm F in human enamel and of 500 ± 70 ppm F in bovine enamel, which is 5–7 times less than the enamel samples used here (2500 ppm)^49,50^. Interestingly, the mean viability of 72% detected on HAP in the present study agrees well with the results of an earlier study. There, the average viability of bacteria in biofilms formed by 15 subjects analogously for 48 h in situ on HAP was 74%^46^. However, the viability on enamel observed here (46%) differs clearly from the findings of the previous study (74%)^46^. This discrepancy in the viability results obtained on enamel samples of different origin underscores the importance of using well-defined and standardized substrates. This might be of great relevance to dental research studies addressing the preventive management of bacterial biofilms.

## Conclusions

No relevant differences were observed with respect to formation kinetics, microstructure, thickness and subject-specificity of the initial biofilm formed on both materials, HAP and enamel. Also at the proteome level, the development of the initial biofilm on HAP follows the formation patterns previously observed on dental enamel. Formation kinetics and morphology of the bacterial biofilm formed on HAP were subject-specific and are similar to that formed on enamel. This also applies to the coverage with microorganisms. However, the bacterial viability on enamel was different in comparison to HAP. Remarkably, the bacterial viability results generated in a previous study under analogous experimental conditions were highly similar to our viability results on HAP but not on enamel^46^. These differences in the viability results obtained on untreated enamel samples underline the comparability of results obtained on synthetic HAP samples and demonstrate that this aspect is particularly important for dental research trials focusing on preventive bacterial biofilm management. In summary, synthetic HAP surfaces can be considered as a full substitute substrate for enamel in dental biofilm studies. For viability studies, synthetic HAP surfaces may even be the preferred substrates as they could be more useful for identifying potential antimicrobial agents. Taken together, the basic phenomena such as pellicle formation, bacterial colonization, and subject-specificity manifest the same way on synthetic HAP as compared to natural enamel surfaces, which shows their suitability for biofilm investigations. Studies for better understanding of dental biofilm formation and on preventive biofilm management with the aim of minimizing oral pathogens will help to improve patient health and thus reduce the burden on the healthcare system.

## Supplementary Information

Supplementary Information.

## Data Availability

The datasets generated and analyzed in this study are available from the corresponding author upon reasonable request.
