# Supplementary material for: Synthetic hydroxyapatite: a perfect substitute for dental enamel in biofilm formation studies
Source: Sci Rep. 2025 Nov 29;15:43379. doi: 10.1038/s41598-025-25634-9 (PMC12690089; doi:10.1038/s41598-025-25634-9)
Supplement: Supplementary file 1 — Supplementary Information. [file 41598_2025_25634_MOESM1_ESM.pdf]

Supporting Information

Synthetic hydroxyapatite – a perfect substitute for dental enamel in biofilm formation studies

Johanna Dudek[1]*, Thomas Faidt[2], Claudia Fecher-Trost[3], Sudharshini Thangamurugan[4], Pardis Bayenat[2], Simone Trautmann[1], Jens Neurohr[2], Anne Holtsch[2], Frank Müller[2], Markus R. Meyer[3], Volkhard Helms[4], Karin Jacobs[2] & Matthias Hannig[1]*

[1] *Clinic of Operative Dentistry, Periodontology and Preventive Dentistry, University Hospital, Saarland University, Homburg/Saar, Germany*

[2] *Experimental Physics and Center for Biophysics, Saarland University, Saarbrücken, Germany*

[3] *Department of Experimental and Clinical Pharmacology and Toxicology, Center for Molecular Signaling (PZMS), Saarland University, Homburg/Saar, Germany*

[4] *Center for Bioinformatics, Saarland University, Saarbrücken, Germany*

**Corresponding authors:*

E-mail: johanna.dudek@uks.eu

E-mail: matthias.hannig@uks.eu

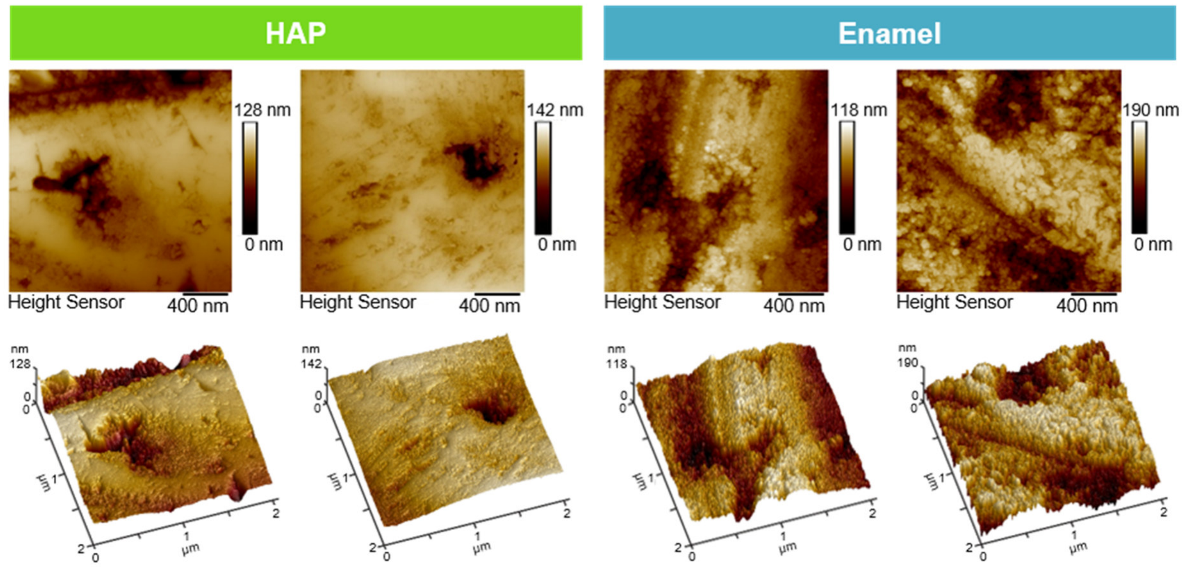

Figure S1. Representative AFM scans of ready for oral exposure (polished and purified) HAP and enamel specimens. The top row shows the 2D representation with colour code for the height of example images for HAP and enamel, while their 3D representation is shown in the bottom row.

Table S1. The topographical properties of ready for oral exposure (polished and purified) HAP and enamel specimens determined by Minkowski functional of AFM scans.[1]

Minkowski functional (normalized* and averaged**)	HAP	Enamel
$\widetilde{W}_0$ (norm. volume) in nm	128 ± 69	108 ± 16
$\widetilde{W}_1$ (norm. surface area)	1.08 ± 0.06	1.45 ± 0.16
$\widetilde{W}_2$ (norm. curvature) in nm^{-1}	$(-2.1 \pm 2.2)\cdot 10^{-4}$	$(-7.6 \pm 8.6)\cdot 10^{-4}$

* The functionals were normalized by the size of the scan window (2 µm × 2 µm).

** Five random spots were scanned for each of three HAP and three enamel specimens. The functionals were averaged over all specimens and spots.

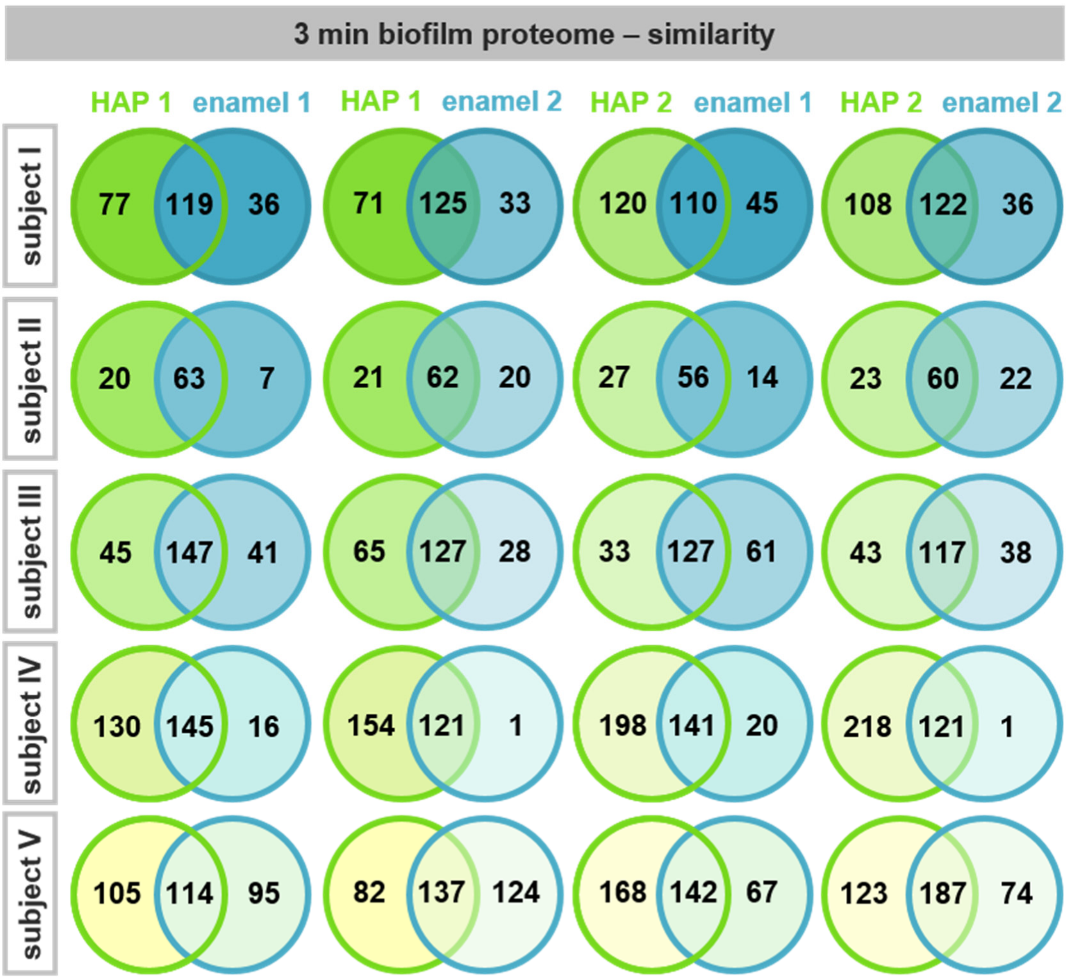

all subjects – p-values

	HAP 1-enamel 1	HAP 1-enamel 2	HAP 2-enamel 1	HAP 2-enamel 2
compared to HAP 1-HAP 2	0.1875	0.0625	0.0625	0.0625
compared to enamel 1-enamel 2	1	1	0.625	0.715

Figure S2. Similarity of proteins commonly found in 3 min biofilms formed on HAP and enamel. Wilcoxon signed rank tests were used to compare the intersection values between HAP 1 and HAP 2 (or enamel 1 and enamel 2) with the intersection values of HAP and enamel replicates (see supplemental figure 2). Null hypothesis: the intersection number of proteins is similar between HAP 1-HAP 2 (or enamel 1-enamel 2) and HAP-enamel. In both cases, the p-values did not show significance. The null hypothesis cannot be rejected. Hence, common proteins between HAP-enamel are similar to the common proteins between HAP 1-HAP 2 or enamel 1-enamel 2. Statistical significance was set at $p < 0.05$.

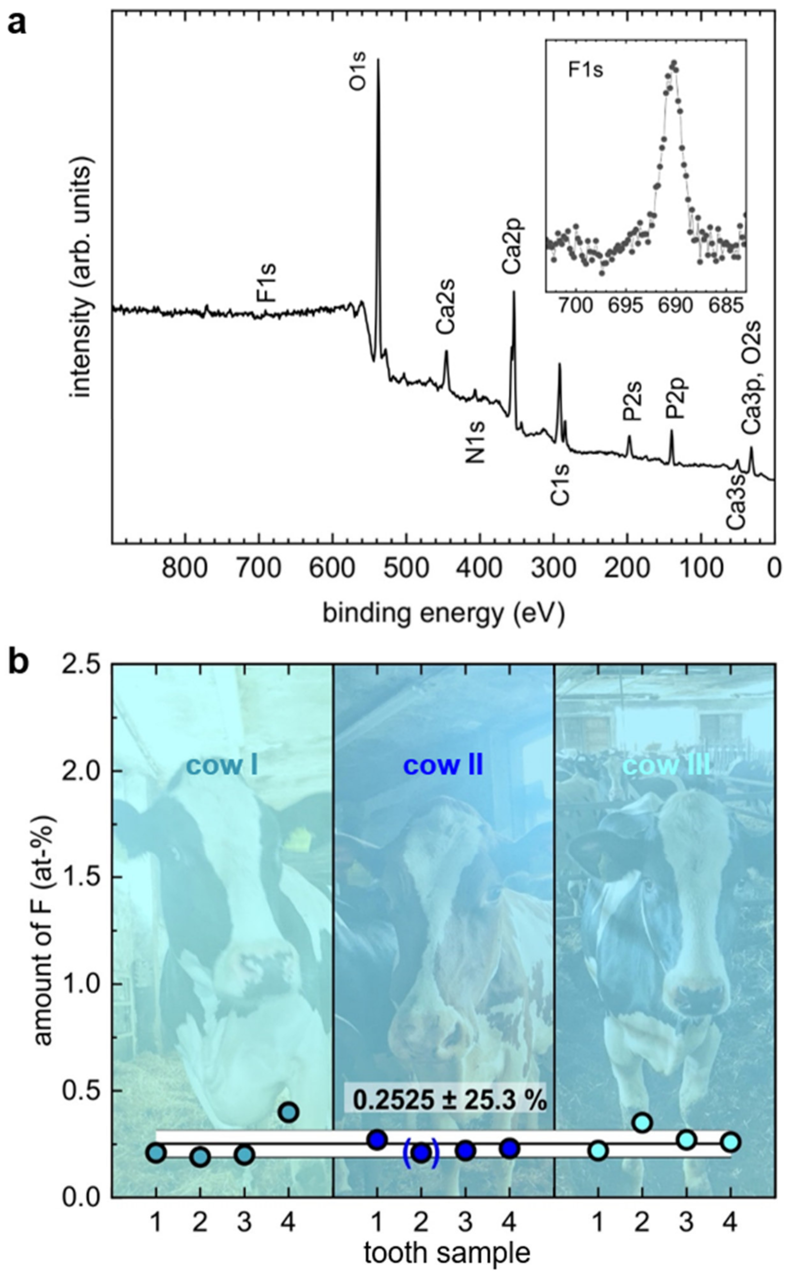

Figure S3. Determination of the fluorine (F) content in the bovine enamel used in this study. (**a**) A typical XPS survey spectrum as taken on bovine enamel used in this study with distinct signals of Ca, P, C, O and traces of N and F. The inset shows the F1s spectrum (with no rescaling of shift in binding energy as caused by charging of the sample). (**b**) The mean value of the relative amount of F (i.e. relative to the overall amount of Ca, P, O and F) is ~ 0.25 at-% with a relative error of about 25 % when averaged over all teeth (the upper value of the ordinate represents the maximum amount of F possible upon hydroxyapatite-fluorapatite transformation.[2] The data point for tooth 2 of cow II is marked with brackets because parts of the dentin were exposed upon grinding of the enamel.

References

1. Schröder-Turk, G. E. *et al.* Minkowski tensor shape analysis of cellular, granular and porous structures. *Adv Mater* **23**, 2535-2553 (2011). https://doi.org:10.1002/adma.201100562

2. de Leeuw, N. H. A computer modelling study of the uptake and segregation of fluoride ions at the hydrated hydroxyapatite (0001) surface: introducing a $Ca_{10}(PO_4)_6(OH)_2$ potential model. *Physical Chemistry Chemical Physics* **6**, 1860-1866 (2004). https://doi.org:10.1039/B313242K
